# Supplementary material for: Digital circuits and neural networks based on acid-base chemistry implemented by robotic fluid handling
Source: Nat Commun. 2023 Jan 30;14:496. doi: 10.1038/s41467-023-36206-8 (PMC9887006; doi:10.1038/s41467-023-36206-8)
Supplement: Supplementary file 1 — Supplementary Information [file 41467_2023_36206_MOESM1_ESM.pdf]

Supplementary Information

Digital Circuits and Neural Networks based on Acid-Base Chemistry implemented by Robotic Fluid Handling

Ahmed A. Agiza[1*], Kady Oakley[1], Jacob K. Rosenstein[1], Brenda M. Rubenstein[1], Eunsuk Kim[1], Marc Riedel[2], and Sherief Reda[1]

[1]Brown University, Providence, RI, USA
[2]University of Minnesota, Minneapolis, MN, USA
[*]ahmed_agiza@brown.edu

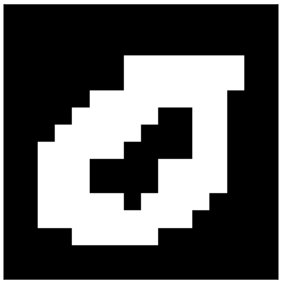

Supplementary Figure 1: An 8×8 example image that results in the wrong classification using acid-base computations. The *in-silico* model generates scores of $\{4.0, 2.0\}$ (before the softmax); consequently, the acid-base model generates pH values of $\{(2.2, 11.8), (2.51, 11.49)\}$, which is correct but indistinguishable by color only.

Supplementary Table 1: Pixel values for the 8×8 example image that results in the wrong classification using acid-base computations.

**Index**	**0**	**1**	**2**	**3**	**4**	**5**	**6**	**7**
**0**	0	0	0	0	0	0	0	0
**1**	0	0	0	1	0	0	0	0
**2**	0	0	0	1	1	0	0	0
**3**	0	0	0	1	0	1	0	0
**4**	0	0	0	1	0	1	0	0
**5**	0	0	0	1	0	1	0	0
**6**	0	0	0	1	1	1	0	0
**7**	0	0	0	0	0	0	0	0

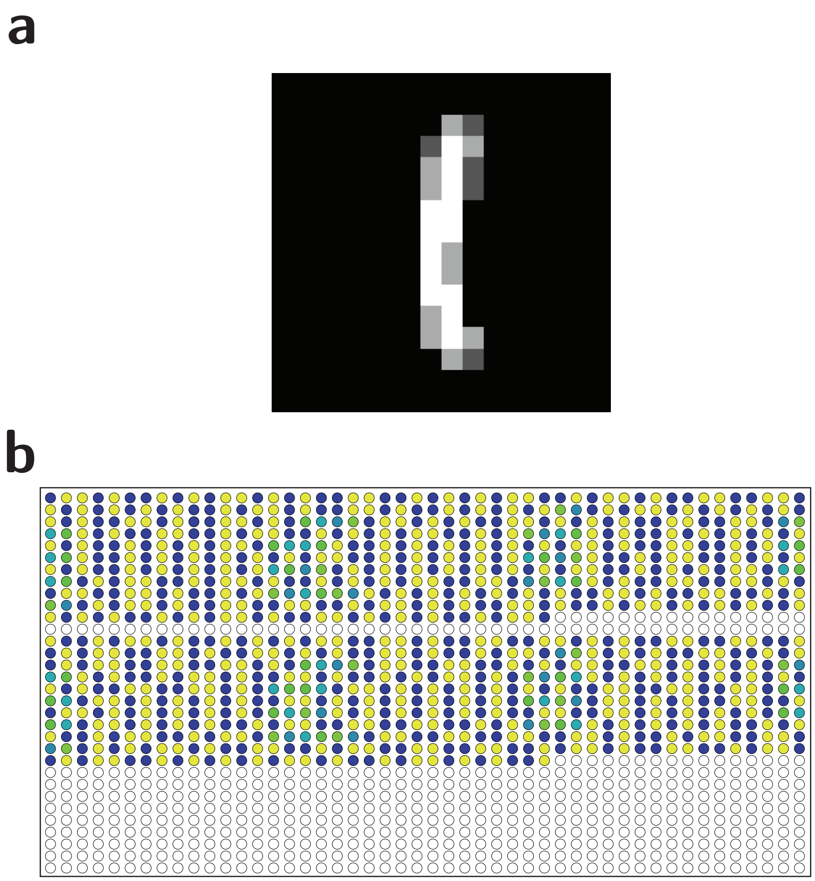

Supplementary Figure 2: Encoding of grayscale image using acid-base dual-rail encoding. **a** Grayscale encoded image to be classified. **b** Encoded image using acids (yellow) and bases (blue). The color intensity and shade of each well correspond to the level of the dilution based on the encoded value following to the pH scale colors shown in Figure 1b.

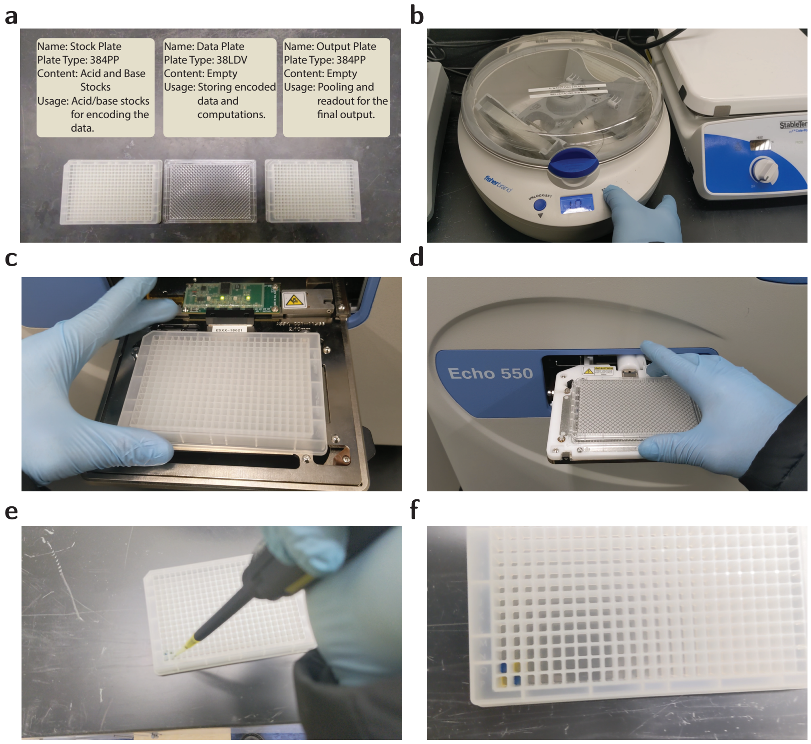

Supplementary Figure 3: Working Setup of the Classification of an 8×8 image. **a** The three plates used for running the experiment. **b** Centrifuging the stock plate. **c** Inserting the stock plate and the data plate into the liquid handler, after which we start the encoding protocol. **d** Retrieving the data plate, followed by centrifugation. Similarly, we insert the data plate and the output plate into the liquid handler, after which we start the pooling protocol. **e** Adding the pH indicator to the pooling plate, followed by centrifugation. **f** Final readout of the network's classification.

Supplementary Note 1. Simulation of Digit Classifier on Microfluidic Board OpenDrop v4

In order to demonstrate that our approach can work on different platforms, we designed a simulation for a similar experiment on OpenDrop v4 [1], a digital microfludics platform that uses electro-wetting technology and is more affordable than the acoustic liquid handler. The board has a 14x8 grid, which we used to encode a 7x7 image with the weights applied as previously described. The simulation then combines the droplets from the positive side of the dual-rail and moves the output to the lower-left cell of the grid, which represents the positive part of the dual-rail neuron's output. Similarly, the negative sides of the dual-rail are combined and moved to the lower-right corner, which represents the negative part of the dual-rail neuron's output. The final output is shown as the pH/color of the dual-pair of the droplet in the lower-left corner of the grid and the droplet in the lower-right corner, where acid-base would indicate +1, and base-acid would indicate -1/0. Supplementary Figure 4 shows the first instance of the simulation with the encoded image and the last instance of the simulation after collecting the results of one neuron. We also ran a full digital logic gate on the physical board, but due to limitations on the volume of the liquid the board can handle, it was not feasible to run the full classification.

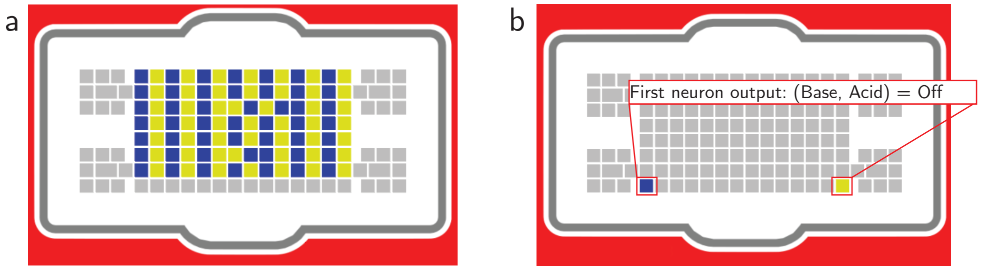

Supplementary Figure 4: Simulation of the Digit Classifier on a Microfluidic Device **a** The encoding of a 7×7 image of the digit one using acids (yellow) and bases (blue) with network weights applied on a microfludic device grid. **b** The final output of the microfluidic simulation of one neuron.

Supplementary Table 2: Weights of neuron 0 in 2-class binary 8×8 image classification.

**Index**	**0**	**1**	**2**	**3**	**4**	**5**	**6**	**7**
**0**	-1	1	-1	1	-1	-1	1	-1
**1**	-1	-1	-1	1	1	1	-1	1
**2**	1	-1	1	1	1	1	1	-1
**3**	-1	1	1	1	-1	1	1	1
**4**	-1	1	1	-1	-1	1	1	-1
**5**	1	1	1	-1	-1	1	1	-1
**6**	1	1	1	1	1	1	-1	1
**7**	-1	1	1	-1	1	-1	-1	-1

Supplementary Table 3: Weights of neuron 1 in 2-class binary 8×8 image classification.

**Index**	**0**	**1**	**2**	**3**	**4**	**5**	**6**	**7**
**0**	1	-1	-1	1	-1	1	1	-1
**1**	1	1	-1	-1	-1	-1	1	-1
**2**	1	-1	-1	-1	-1	-1	-1	-1
**3**	1	-1	-1	-1	1	-1	-1	-1
**4**	1	-1	-1	-1	1	-1	-1	-1
**5**	-1	-1	-1	1	-1	-1	-1	1
**6**	-1	-1	-1	-1	-1	-1	1	1
**7**	1	-1	1	-1	1	-1	1	1

Supplementary Table 4: Weights of neuron 0 in 2-class binary 12×12 image classification.

**Index**	**0**	**1**	**2**	**3**	**4**	**5**	**6**	**7**	**8**	**9**	**10**	**11**
**0**	-1	1	-1	-1	-1	1	1	1	-1	-1	-1	1
**1**	1	1	1	-1	-1	-1	-1	-1	-1	1	-1	-1
**2**	1	1	-1	1	-1	1	-1	1	1	-1	1	-1
**3**	1	1	-1	1	1	1	1	1	1	1	-1	-1
**4**	-1	-1	-1	1	1	1	-1	-1	1	1	1	-1
**5**	1	-1	1	1	1	-1	-1	-1	1	1	1	1
**6**	-1	-1	1	1	1	-1	-1	-1	1	1	-1	1
**7**	1	-1	1	1	1	-1	-1	1	1	1	1	1
**8**	-1	-1	1	1	1	-1	1	1	1	-1	1	-1
**9**	1	1	1	1	1	1	1	1	-1	-1	-1	-1
**10**	-1	-1	-1	-1	1	1	-1	1	-1	-1	-1	-1
**11**	-1	-1	1	1	1	1	-1	-1	1	1	-1	1

Supplementary Table 5: Weights of neuron 1 in 2-class binary 12×12 image classification.

**Index**	**0**	**1**	**2**	**3**	**4**	**5**	**6**	**7**	**8**	**9**	**10**	**11**
**0**	-1	1	-1	-1	1	1	1	1	1	1	1	-1
**1**	1	-1	-1	1	1	1	1	-1	-1	1	-1	-1
**2**	-1	1	-1	-1	-1	-1	-1	-1	1	1	-1	1
**3**	1	-1	1	1	-1	-1	-1	-1	-1	-1	-1	1
**4**	-1	1	1	-1	-1	-1	1	-1	-1	-1	1	1
**5**	1	-1	-1	-1	-1	-1	1	-1	-1	-1	-1	-1
**6**	-1	-1	-1	-1	-1	1	1	-1	-1	-1	-1	-1
**7**	-1	1	-1	-1	-1	1	1	-1	-1	-1	-1	-1
**8**	-1	-1	-1	-1	-1	1	-1	-1	-1	-1	-1	1
**9**	1	-1	1	-1	-1	-1	-1	-1	1	1	-1	-1
**10**	1	-1	1	1	1	-1	1	1	-1	1	-1	1
**11**	1	1	1	-1	1	-1	1	1	-1	-1	1	-1

Supplementary Table 6: Weights of neuron 0 in 2-class binary 16×16 image classification.

**Index**	**0**	**1**	**2**	**3**	**4**	**5**	**6**	**7**	**8**	**9**	**10**	**11**	**12**	**13**	**14**	**15**
**0**	-1	-1	1	-1	-1	1	-1	-1	-1	1	-1	1	-1	1	1	1
**1**	-1	-1	-1	1	-1	-1	-1	-1	-1	-1	-1	1	-1	1	1	1
**2**	-1	-1	-1	-1	1	-1	-1	1	-1	-1	1	-1	-1	-1	1	1
**3**	1	-1	-1	-1	1	1	1	1	1	1	-1	1	-1	-1	-1	1
**4**	1	1	-1	1	-1	1	1	1	-1	1	1	1	1	1	-1	-1
**5**	1	1	1	1	1	-1	1	1	1	1	1	1	1	1	1	-1
**6**	1	1	1	-1	-1	1	1	-1	-1	-1	1	1	1	1	1	1
**7**	-1	1	1	1	1	1	1	-1	-1	-1	-1	1	1	1	1	-1
**8**	-1	-1	1	1	1	1	1	-1	-1	-1	-1	1	1	1	1	-1
**9**	1	-1	1	1	1	1	-1	-1	-1	-1	1	1	1	1	-1	-1
**10**	-1	-1	-1	1	1	1	1	-1	-1	1	1	1	-1	1	-1	-1
**11**	-1	1	1	1	1	1	1	-1	1	1	1	1	-1	1	1	1
**12**	1	-1	-1	1	1	1	1	1	1	1	-1	-1	-1	-1	-1	-1
**13**	1	1	-1	-1	1	1	1	1	1	-1	-1	-1	-1	1	1	-1
**14**	1	1	1	-1	1	-1	-1	1	-1	1	-1	1	1	1	-1	1
**15**	-1	-1	-1	-1	-1	-1	1	-1	1	-1	1	-1	-1	1	1	-1

Supplementary Table 7: Weights of neuron 1 in 2-class binary 16×16 image classification.

**Index**	**0**	**1**	**2**	**3**	**4**	**5**	**6**	**7**	**8**	**9**	**10**	**11**	**12**	**13**	**14**	**15**
**0**	-1	1	1	1	-1	1	-1	1	1	-1	1	1	-1	1	-1	1
**1**	-1	1	-1	1	-1	-1	-1	1	-1	1	-1	-1	1	-1	-1	1
**2**	1	-1	1	1	1	1	1	-1	1	-1	1	-1	1	-1	-1	-1
**3**	1	1	-1	1	-1	1	-1	-1	-1	-1	-1	1	-1	1	-1	-1
**4**	-1	1	-1	1	-1	-1	-1	-1	-1	-1	-1	-1	-1	-1	1	-1
**5**	-1	1	1	1	-1	1	-1	-1	-1	1	-1	-1	-1	-1	-1	1
**6**	-1	1	-1	-1	-1	-1	-1	-1	1	1	-1	-1	-1	-1	-1	1
**7**	1	1	-1	-1	-1	-1	-1	1	1	1	-1	-1	-1	-1	1	1
**8**	-1	1	-1	-1	-1	-1	-1	1	1	-1	-1	-1	-1	-1	-1	-1
**9**	1	-1	1	-1	-1	-1	-1	1	1	-1	-1	-1	-1	-1	-1	1
**10**	1	1	-1	-1	-1	-1	-1	1	1	-1	-1	-1	1	-1	-1	-1
**11**	-1	-1	1	-1	-1	-1	-1	-1	-1	-1	-1	-1	1	-1	-1	-1
**12**	-1	1	-1	-1	1	-1	-1	1	-1	-1	-1	1	1	1	-1	1
**13**	1	-1	-1	1	-1	-1	-1	-1	-1	1	1	-1	1	-1	1	-1
**14**	1	-1	1	1	1	1	1	-1	-1	-1	1	-1	1	1	-1	1
**15**	1	-1	1	-1	1	1	-1	1	-1	-1	-1	-1	1	-1	-1	-1

Supplementary Table 8: Weights of neuron 0 in 2-class binary 28×28 image classification.

**Index**	**0**	**1**	**2**	**3**	**4**	**5**	**6**	**7**	**8**	**9**	**10**	**11**	**12**	**13**	**14**	**15**	**16**	**17**	**18**	**19**	**20**	**21**	**22**	**23**	**24**	**25**	**26**	**27**
**0**	1	-1	1	-1	-1	-1	-1	1	1	1	-1	1	-1	1	1	-1	1	-1	-1	-1	1	1	-1	-1	-1	1	-1	-1
**1**	1	-1	-1	-1	1	-1	1	-1	1	-1	1	-1	-1	1	-1	-1	-1	-1	-1	1	1	1	-1	-1	1	-1	-1	-1
**2**	-1	-1	1	-1	-1	-1	1	-1	1	-1	-1	-1	1	-1	-1	-1	-1	-1	1	1	-1	-1	1	-1	-1	1	1	-1
**3**	-1	-1	1	-1	1	-1	1	-1	-1	1	1	-1	1	1	-1	-1	-1	-1	1	-1	-1	1	1	1	-1	1	-1	-1
**4**	1	1	-1	1	1	-1	-1	1	1	-1	-1	-1	-1	1	-1	1	-1	-1	-1	1	-1	-1	-1	-1	-1	1	-1	-1
**5**	-1	1	1	-1	1	-1	-1	-1	-1	-1	1	1	1	-1	1	-1	-1	1	1	-1	1	1	-1	-1	-1	-1	1	1
**6**	-1	1	1	-1	1	1	-1	1	-1	1	1	1	-1	-1	1	1	1	-1	1	-1	1	-1	-1	1	1	1	1	-1
**7**	1	-1	1	1	1	1	-1	-1	-1	1	1	1	1	1	1	-1	1	1	1	1	-1	-1	1	-1	1	-1	1	1
**8**	1	1	1	-1	-1	-1	-1	-1	-1	1	-1	1	1	-1	-1	1	-1	1	1	1	1	1	1	1	1	-1	-1	1
**9**	1	1	1	1	-1	1	1	-1	1	-1	-1	1	-1	-1	1	-1	-1	1	-1	1	1	1	1	-1	-1	1	1	1
**10**	-1	1	1	-1	1	-1	-1	-1	-1	1	1	1	1	-1	-1	-1	-1	1	-1	1	1	1	1	1	1	-1	1	-1
**11**	1	-1	1	1	-1	1	-1	-1	1	1	-1	1	1	-1	-1	-1	-1	1	1	1	1	1	1	1	-1	1	1	1
**12**	-1	-1	-1	1	-1	-1	-1	1	1	1	1	1	-1	-1	-1	-1	-1	-1	1	1	1	1	1	1	-1	1	1	-1
**13**	-1	1	1	-1	1	1	1	1	1	1	1	1	-1	-1	1	-1	-1	-1	-1	1	1	1	1	1	1	1	-1	1
**14**	1	-1	-1	-1	1	1	-1	1	1	1	1	1	-1	-1	-1	-1	-1	-1	1	1	1	1	1	1	1	1	-1	1
**15**	1	-1	1	-1	-1	1	1	-1	1	1	1	1	-1	-1	-1	-1	-1	1	1	1	1	1	1	1	1	1	1	-1
**16**	1	-1	-1	1	1	1	1	1	1	1	1	-1	1	1	-1	-1	1	1	1	1	1	1	1	-1	1	1	1	1
**17**	-1	-1	-1	-1	1	1	1	1	1	1	1	-1	-1	-1	-1	1	-1	1	1	1	1	1	1	1	-1	1	-1	-1
**18**	-1	-1	1	1	1	1	-1	1	1	-1	1	-1	-1	-1	1	-1	1	1	1	1	-1	1	1	1	-1	-1	-1	-1
**19**	1	1	1	1	1	1	1	1	1	-1	1	1	-1	-1	-1	-1	1	1	-1	1	1	1	-1	1	1	1	1	-1
**20**	-1	-1	-1	1	1	1	-1	1	1	1	1	1	-1	1	-1	1	1	-1	1	1	-1	-1	1	-1	1	1	1	-1
**21**	1	-1	1	-1	-1	-1	-1	1	-1	-1	1	1	1	1	1	1	-1	-1	1	1	-1	-1	-1	-1	-1	1	-1	-1
**22**	-1	-1	1	-1	1	-1	-1	1	1	-1	1	1	1	1	-1	1	-1	1	-1	-1	1	-1	1	1	1	1	-1	-1
**23**	1	-1	-1	-1	-1	1	-1	-1	1	1	1	1	1	1	1	-1	1	-1	1	1	-1	-1	1	1	1	-1	1	1
**24**	1	-1	-1	1	-1	1	-1	1	-1	-1	-1	-1	-1	1	1	-1	-1	1	1	-1	-1	-1	1	-1	1	1	-1	-1
**25**	1	-1	1	-1	-1	-1	-1	-1	-1	-1	1	-1	-1	-1	-1	1	-1	1	1	-1	-1	-1	1	1	-1	-1	1	-1
**26**	-1	-1	-1	-1	-1	1	1	-1	1	-1	-1	1	-1	-1	-1	-1	1	1	1	-1	-1	1	1	-1	-1	-1	-1	-1
**27**	-1	-1	1	-1	1	-1	1	1	-1	1	-1	-1	1	1	-1	-1	-1	-1	-1	1	1	1	1	-1	1	-1	-1	-1

Supplementary Table 9: Weights of neuron 1 in 2-class binary 28×28 image classification.

**Index**	**0**	**1**	**2**	**3**	**4**	**5**	**6**	**7**	**8**	**9**	**10**	**11**	**12**	**13**	**14**	**15**	**16**	**17**	**18**	**19**	**20**	**21**	**22**	**23**	**24**	**25**	**26**	**27**
**0**	1	-1	1	-1	1	1	-1	-1	-1	1	1	1	-1	-1	1	-1	-1	-1	-1	1	-1	1	1	-1	-1	1	1	-1
**1**	1	1	-1	1	1	1	-1	-1	1	-1	1	-1	-1	1	1	1	1	-1	1	1	1	-1	-1	-1	-1	1	-1	-1
**2**	1	-1	1	1	1	1	1	1	-1	1	-1	1	1	-1	-1	1	-1	1	-1	-1	1	1	-1	-1	-1	-1	-1	-1
**3**	1	1	-1	1	-1	1	-1	1	-1	1	1	1	-1	1	1	-1	-1	1	1	1	-1	-1	1	1	1	-1	-1	1
**4**	-1	1	-1	1	1	1	-1	-1	-1	1	1	-1	1	1	-1	1	1	-1	-1	1	-1	1	1	-1	1	-1	1	1
**5**	-1	-1	-1	1	-1	1	1	-1	-1	1	1	1	-1	-1	1	1	-1	-1	1	-1	1	1	-1	1	-1	1	-1	-1
**6**	1	-1	1	1	-1	1	1	1	-1	1	1	-1	1	-1	-1	1	-1	-1	-1	-1	1	-1	1	1	-1	-1	-1	-1
**7**	-1	-1	1	1	1	-1	1	1	-1	1	-1	1	-1	-1	1	1	-1	-1	-1	-1	-1	-1	1	-1	-1	1	1	1
**8**	1	1	-1	1	1	1	1	-1	-1	-1	-1	-1	-1	-1	-1	1	1	-1	1	-1	-1	-1	-1	-1	1	1	-1	-1
**9**	1	1	1	1	1	1	-1	1	-1	-1	-1	-1	-1	1	-1	1	-1	1	-1	-1	-1	-1	1	1	1	-1	-1	1
**10**	-1	-1	-1	1	-1	-1	1	-1	-1	1	1	-1	-1	-1	-1	1	1	-1	1	-1	-1	-1	1	-1	1	1	-1	1
**11**	-1	1	-1	-1	-1	-1	-1	-1	1	-1	-1	-1	-1	-1	1	1	1	-1	-1	-1	-1	1	-1	-1	-1	-1	-1	1
**12**	-1	1	-1	1	1	-1	-1	-1	-1	-1	-1	-1	-1	1	1	1	1	-1	-1	-1	-1	1	-1	-1	-1	1	-1	-1
**13**	-1	1	1	-1	-1	1	-1	-1	-1	-1	-1	-1	-1	1	1	1	-1	1	-1	-1	1	-1	-1	-1	-1	-1	1	1
**14**	1	1	-1	1	1	1	-1	-1	-1	-1	-1	-1	-1	1	1	1	1	-1	-1	-1	-1	-1	-1	-1	1	-1	-1	1
**15**	1	-1	-1	1	1	-1	-1	-1	-1	-1	-1	-1	-1	1	1	1	1	-1	-1	-1	-1	1	-1	-1	1	-1	-1	1
**16**	-1	1	-1	-1	1	-1	-1	1	-1	-1	-1	-1	1	1	1	1	-1	-1	-1	-1	-1	-1	-1	1	-1	1	1	1
**17**	-1	-1	1	1	-1	-1	-1	-1	-1	-1	-1	-1	1	-1	1	-1	1	-1	-1	-1	-1	1	-1	1	-1	1	-1	1
**18**	1	1	-1	1	1	1	-1	-1	-1	-1	1	1	1	1	1	1	-1	-1	-1	-1	-1	-1	-1	-1	1	1	1	1
**19**	-1	-1	1	-1	-1	-1	-1	-1	-1	-1	-1	1	-1	1	1	-1	-1	-1	1	-1	1	-1	-1	-1	-1	1	1	-1
**20**	-1	1	1	-1	1	1	1	1	-1	-1	-1	-1	-1	-1	1	-1	-1	-1	1	1	-1	-1	-1	1	-1	1	1	1
**21**	-1	1	-1	-1	-1	-1	1	-1	1	1	-1	-1	1	-1	1	-1	1	-1	1	-1	1	1	-1	1	-1	1	1	1
**22**	1	-1	1	-1	1	-1	-1	-1	1	-1	-1	1	-1	-1	1	-1	-1	1	-1	-1	1	1	1	1	1	-1	1	-1
**23**	-1	1	-1	-1	1	1	-1	1	1	-1	-1	-1	-1	-1	-1	1	-1	1	-1	1	1	1	1	1	-1	1	-1	1
**24**	-1	1	1	1	-1	-1	1	1	-1	-1	1	1	-1	1	-1	1	-1	1	-1	-1	-1	1	-1	-1	-1	-1	-1	1
**25**	-1	1	1	1	1	1	1	1	1	-1	-1	1	-1	-1	1	-1	1	1	1	1	-1	1	-1	-1	1	-1	1	-1
**26**	1	1	1	1	1	1	-1	1	1	1	-1	-1	-1	-1	1	1	1	-1	1	-1	1	1	1	1	-1	-1	1	-1
**27**	-1	1	1	1	-1	1	-1	1	-1	-1	1	-1	-1	-1	1	-1	-1	1	-1	-1	1	-1	-1	1	1	-1	-1	-1

Supplementary Table 10: Weights of neuron 0 in 3-class binary 8×8 image classification.

**Index**	**0**	**1**	**2**	**3**	**4**	**5**	**6**	**7**
**0**	-1	-1	1	1	-1	1	1	-1
**1**	-1	-1	-1	-1	1	1	1	1
**2**	1	-1	1	1	1	1	1	1
**3**	1	1	1	1	-1	-1	1	1
**4**	-1	1	1	-1	-1	-1	1	-1
**5**	1	-1	1	-1	-1	1	-1	-1
**6**	1	-1	1	1	1	1	-1	1
**7**	-1	1	1	-1	-1	1	1	1

Supplementary Table 11: Weights of neuron 1 in 3-class binary 8×8 image classification.

**Index**	**0**	**1**	**2**	**3**	**4**	**5**	**6**	**7**
**0**	1	1	1	-1	-1	-1	1	1
**1**	1	1	-1	-1	-1	1	1	-1
**2**	1	-1	-1	-1	1	-1	-1	1
**3**	1	-1	-1	1	1	-1	-1	-1
**4**	1	-1	-1	-1	1	-1	-1	-1
**5**	-1	-1	-1	-1	-1	-1	-1	-1
**6**	1	1	-1	1	1	-1	-1	1
**7**	1	-1	1	1	-1	1	1	1

Supplementary Table 12: Weights of neuron 2 in 3-class binary 8×8 image classification.

**Index**	**0**	**1**	**2**	**3**	**4**	**5**	**6**	**7**
**0**	1	-1	1	-1	-1	1	-1	1
**1**	1	1	1	1	1	1	-1	1
**2**	1	1	1	-1	-1	-1	-1	-1
**3**	-1	-1	-1	-1	1	1	-1	1
**4**	-1	1	-1	1	1	1	-1	1
**5**	1	1	1	1	1	1	1	1
**6**	-1	1	1	-1	-1	1	1	1
**7**	-1	-1	-1	-1	1	-1	1	-1

Supplementary Table 13: Weights of neuron 0 in 3-class binary 12×12 image classification.

**Index**	**0**	**1**	**2**	**3**	**4**	**5**	**6**	**7**	**8**	**9**	**10**	**11**
**0**	1	-1	1	-1	-1	1	-1	1	-1	-1	1	1
**1**	1	1	1	-1	-1	-1	-1	-1	-1	1	-1	1
**2**	-1	1	-1	-1	1	-1	1	-1	1	-1	1	-1
**3**	1	-1	-1	-1	-1	-1	1	1	1	1	1	1
**4**	1	1	1	1	1	1	1	1	1	1	1	1
**5**	1	-1	1	1	1	1	-1	-1	1	1	1	-1
**6**	1	1	1	1	1	-1	-1	-1	1	1	1	-1
**7**	1	-1	1	1	-1	-1	-1	-1	1	1	-1	-1
**8**	1	-1	-1	1	1	-1	-1	1	-1	-1	-1	-1
**9**	1	-1	-1	-1	1	1	1	1	-1	-1	-1	1
**10**	1	1	-1	-1	1	1	1	1	-1	-1	1	-1
**11**	-1	1	1	1	-1	1	-1	1	1	-1	1	1

Supplementary Table 14: Weights of neuron 1 in 3-class binary 12×12 image classification.

**Index**	**0**	**1**	**2**	**3**	**4**	**5**	**6**	**7**	**8**	**9**	**10**	**11**
**0**	1	1	1	1	-1	1	-1	-1	1	1	1	-1
**1**	1	1	-1	-1	-1	-1	-1	-1	1	-1	1	1
**2**	-1	-1	-1	-1	-1	-1	-1	-1	1	1	1	1
**3**	1	1	-1	-1	-1	-1	-1	-1	1	-1	1	1
**4**	1	1	-1	-1	-1	1	1	-1	-1	-1	-1	-1
**5**	1	-1	-1	-1	-1	1	1	-1	-1	-1	1	1
**6**	-1	1	-1	-1	-1	1	1	-1	-1	-1	-1	-1
**7**	-1	1	-1	-1	-1	1	1	-1	-1	-1	-1	1
**8**	1	-1	-1	-1	-1	1	-1	-1	-1	-1	-1	1
**9**	1	1	1	1	-1	1	1	-1	-1	-1	-1	1
**10**	1	1	1	1	1	1	1	-1	1	-1	1	-1
**11**	1	-1	1	1	1	1	1	1	1	-1	1	1

Supplementary Table 15: Weights of neuron 2 in 3-class binary 12×12 image classification.

**Index**	**0**	**1**	**2**	**3**	**4**	**5**	**6**	**7**	**8**	**9**	**10**	**11**
**0**	1	-1	-1	1	-1	1	-1	1	1	-1	-1	-1
**1**	-1	1	-1	1	1	1	1	1	-1	1	-1	1
**2**	-1	-1	1	1	1	1	1	1	-1	1	-1	-1
**3**	-1	1	1	1	1	1	1	-1	-1	-1	1	-1
**4**	1	1	-1	-1	-1	-1	-1	1	-1	-1	1	-1
**5**	1	1	-1	-1	-1	-1	1	1	-1	-1	-1	1
**6**	1	-1	-1	-1	1	1	1	1	1	-1	-1	1
**7**	1	1	1	1	1	1	1	1	1	1	1	1
**8**	1	1	1	1	1	1	1	1	1	1	1	-1
**9**	-1	1	1	1	-1	-1	-1	-1	1	1	1	-1
**10**	1	1	1	-1	-1	-1	1	1	1	1	-1	-1
**11**	-1	-1	-1	-1	-1	1	-1	1	-1	-1	-1	1

Supplementary Table 16: Weights of neuron 0 in 3-class binary 16×16 image classification.

**Index**	**0**	**1**	**2**	**3**	**4**	**5**	**6**	**7**	**8**	**9**	**10**	**11**	**12**	**13**	**14**	**15**
**0**	-1	-1	-1	1	1	1	1	1	1	1	1	-1	-1	-1	1	1
**1**	1	-1	-1	1	1	-1	-1	1	-1	-1	1	-1	-1	-1	1	1
**2**	1	-1	1	1	-1	1	1	1	1	1	1	-1	-1	-1	-1	1
**3**	1	1	1	-1	-1	-1	-1	-1	1	1	-1	-1	1	1	1	-1
**4**	-1	1	-1	-1	-1	-1	-1	1	1	1	1	1	-1	1	1	1
**5**	-1	1	-1	1	-1	1	1	1	1	1	1	1	1	1	-1	1
**6**	1	1	1	1	1	1	1	1	-1	-1	1	1	1	1	1	1
**7**	1	1	1	1	1	1	1	1	-1	-1	-1	1	1	1	1	-1
**8**	1	-1	1	1	1	1	-1	-1	-1	-1	-1	1	1	1	1	-1
**9**	1	-1	1	1	1	1	-1	-1	-1	-1	-1	1	-1	-1	-1	1
**10**	1	-1	-1	-1	1	1	-1	-1	-1	-1	-1	1	1	-1	1	1
**11**	-1	1	-1	-1	-1	1	1	-1	-1	1	1	1	-1	-1	-1	1
**12**	-1	-1	-1	-1	-1	1	1	1	1	1	1	-1	-1	-1	1	-1
**13**	1	-1	-1	-1	-1	1	1	1	1	1	-1	-1	-1	1	-1	-1
**14**	-1	1	-1	1	-1	-1	1	1	1	1	1	-1	1	-1	1	1
**15**	1	-1	1	1	1	1	-1	1	-1	-1	-1	1	1	1	1	-1

Supplementary Table 17: Weights of neuron 1 in 3-class binary 16 $\times$ 16 image classification.

**Index**	**0**	**1**	**2**	**3**	**4**	**5**	**6**	**7**	**8**	**9**	**10**	**11**	**12**	**13**	**14**	**15**
**0**	1	1	-1	1	1	1	-1	1	-1	1	-1	-1	1	-1	1	1
**1**	1	1	1	-1	-1	-1	-1	-1	-1	-1	-1	1	-1	1	-1	1
**2**	-1	1	1	1	-1	-1	-1	-1	1	-1	1	1	1	1	1	-1
**3**	1	-1	1	-1	-1	-1	-1	-1	-1	-1	-1	1	1	-1	1	1
**4**	1	-1	1	-1	-1	-1	-1	-1	-1	1	-1	-1	1	1	1	1
**5**	1	-1	-1	-1	-1	-1	-1	1	1	1	-1	-1	-1	-1	1	-1
**6**	1	1	1	-1	-1	-1	-1	1	1	-1	-1	-1	-1	-1	1	1
**7**	1	1	1	1	-1	-1	1	1	1	-1	-1	-1	-1	-1	-1	1
**8**	-1	1	1	-1	-1	-1	-1	1	1	1	-1	-1	-1	-1	-1	-1
**9**	1	1	-1	-1	-1	-1	-1	1	1	-1	-1	-1	-1	-1	-1	1
**10**	-1	1	1	-1	-1	-1	-1	-1	1	-1	-1	-1	-1	-1	-1	1
**11**	1	1	-1	-1	-1	-1	-1	1	-1	-1	-1	-1	-1	-1	-1	-1
**12**	1	-1	1	-1	-1	-1	-1	-1	-1	-1	-1	-1	-1	1	-1	-1
**13**	-1	1	1	1	1	1	1	1	1	1	-1	-1	-1	1	1	-1
**14**	-1	1	1	1	1	-1	1	1	-1	1	-1	1	-1	1	1	-1
**15**	1	1	1	1	1	-1	-1	-1	1	-1	1	1	-1	-1	1	1

Supplementary Table 18: Weights of neuron 2 in 3-class binary 16×16 image classification.

**Index**	**0**	**1**	**2**	**3**	**4**	**5**	**6**	**7**	**8**	**9**	**10**	**11**	**12**	**13**	**14**	**15**
**0**	1	-1	-1	1	1	1	-1	1	-1	-1	1	-1	1	-1	-1	-1
**1**	1	1	1	1	-1	-1	1	1	-1	-1	-1	1	1	1	1	-1
**2**	-1	-1	1	-1	1	-1	1	1	1	1	1	-1	-1	-1	1	-1
**3**	1	1	-1	1	1	1	1	1	1	1	1	-1	1	-1	1	-1
**4**	-1	1	1	1	1	1	1	1	-1	-1	-1	1	-1	1	-1	-1
**5**	1	1	1	1	-1	-1	-1	-1	-1	-1	1	-1	-1	-1	-1	-1
**6**	1	1	-1	-1	-1	-1	-1	-1	-1	1	-1	-1	-1	-1	1	-1
**7**	-1	-1	-1	-1	-1	-1	-1	-1	1	1	1	-1	-1	-1	-1	-1
**8**	1	-1	-1	-1	-1	1	1	1	1	1	1	1	-1	-1	-1	1
**9**	-1	1	1	1	1	1	1	1	1	1	1	-1	1	1	1	1
**10**	-1	-1	1	1	1	1	1	1	1	-1	1	1	1	1	1	-1
**11**	1	1	1	1	1	1	1	1	1	1	1	1	1	1	1	-1
**12**	1	1	1	1	1	-1	-1	1	-1	-1	1	1	1	1	1	1
**13**	1	1	1	-1	1	-1	-1	-1	-1	-1	1	1	1	1	-1	-1
**14**	-1	1	-1	1	-1	1	1	1	-1	1	1	-1	1	1	1	1
**15**	-1	1	1	-1	-1	1	-1	-1	-1	1	-1	1	-1	1	-1	-1

Supplementary Table 19: Weights of neuron 0 in 3-class binary 28×28 image classification.

**Index**	**0**	**1**	**2**	**3**	**4**	**5**	**6**	**7**	**8**	**9**	**10**	**11**	**12**	**13**	**14**	**15**	**16**	**17**	**18**	**19**	**20**	**21**	**22**	**23**	**24**	**25**	**26**	**27**
**0**	-1	-1	-1	-1	1	-1	1	-1	1	-1	1	-1	-1	1	-1	1	-1	1	1	-1	-1	1	-1	-1	-1	1	1	1
**1**	-1	-1	1	1	1	1	-1	1	1	-1	1	1	-1	1	-1	1	1	1	1	1	1	-1	-1	1	-1	1	1	-1
**2**	-1	-1	1	-1	1	1	1	1	-1	-1	-1	1	1	-1	-1	1	1	-1	-1	1	-1	-1	1	1	-1	-1	1	-1
**3**	-1	-1	-1	1	-1	1	-1	-1	1	-1	1	-1	-1	-1	-1	-1	1	-1	1	-1	1	-1	1	-1	1	-1	1	-1
**4**	-1	1	1	-1	-1	-1	-1	1	-1	-1	-1	1	1	-1	1	-1	-1	1	1	-1	-1	1	-1	1	1	1	-1	1
**5**	1	1	1	-1	-1	1	-1	-1	1	-1	1	-1	-1	1	1	-1	-1	1	-1	-1	1	-1	1	-1	1	-1	-1	-1
**6**	-1	1	1	1	1	1	-1	1	-1	-1	-1	-1	1	-1	-1	-1	1	1	1	1	-1	1	-1	-1	1	1	1	1
**7**	1	-1	-1	-1	1	1	-1	-1	-1	-1	-1	-1	1	1	1	1	1	1	-1	1	1	1	1	1	1	1	-1	-1
**8**	-1	-1	-1	-1	1	-1	-1	1	-1	1	-1	-1	1	-1	1	1	-1	1	1	-1	-1	-1	1	-1	1	1	-1	-1
**9**	-1	-1	1	-1	1	1	-1	1	-1	1	-1	1	-1	1	-1	1	-1	1	1	1	1	-1	1	1	1	1	1	-1
**10**	1	-1	1	-1	1	-1	1	1	1	1	1	1	1	1	1	-1	1	-1	1	1	1	1	-1	1	1	-1	-1	-1
**11**	-1	1	-1	1	1	1	-1	1	-1	1	1	1	1	1	1	-1	-1	1	1	1	-1	1	1	1	1	-1	-1	1
**12**	-1	1	1	-1	1	1	1	1	1	1	1	1	1	1	-1	-1	-1	1	-1	1	1	1	1	1	1	-1	-1	1
**13**	1	1	-1	1	1	1	1	1	1	1	1	1	-1	-1	-1	-1	-1	-1	1	1	-1	1	1	1	-1	1	1	-1
**14**	1	-1	1	1	1	1	1	1	1	1	1	1	-1	-1	-1	-1	-1	-1	-1	1	1	1	1	1	-1	1	-1	1
**15**	1	1	1	1	-1	1	1	1	1	1	1	-1	-1	-1	-1	-1	-1	-1	-1	1	-1	1	1	1	1	1	1	1
**16**	1	1	1	1	-1	1	1	1	1	1	1	-1	-1	-1	-1	-1	-1	-1	-1	-1	-1	-1	-1	1	1	-1	-1	1
**17**	-1	1	1	-1	-1	-1	1	1	-1	1	1	-1	-1	-1	-1	-1	-1	1	-1	-1	-1	1	-1	-1	-1	-1	1	-1
**18**	-1	1	1	-1	-1	-1	1	1	1	1	1	-1	-1	-1	-1	-1	-1	-1	1	1	-1	1	-1	1	1	-1	1	1
**19**	-1	1	-1	1	-1	-1	-1	1	-1	1	1	1	-1	-1	-1	-1	-1	-1	1	1	-1	1	-1	-1	-1	-1	1	-1
**20**	-1	1	-1	-1	1	1	1	1	-1	1	1	1	-1	1	-1	1	1	-1	-1	-1	1	-1	-1	-1	-1	1	1	1
**21**	-1	1	-1	-1	-1	-1	1	-1	1	-1	1	1	1	-1	-1	1	1	-1	-1	-1	-1	-1	-1	-1	-1	1	1	-1
**22**	-1	1	1	-1	-1	-1	-1	-1	-1	1	1	1	1	1	1	1	-1	1	1	-1	1	-1	1	-1	-1	1	1	1
**23**	1	1	-1	1	-1	1	-1	-1	-1	-1	1	1	1	1	1	1	1	-1	-1	-1	1	-1	-1	-1	-1	-1	1	-1
**24**	-1	-1	-1	1	1	1	-1	-1	-1	-1	1	-1	1	1	1	1	1	-1	-1	1	-1	-1	1	1	1	-1	1	-1
**25**	-1	-1	1	1	1	1	-1	1	-1	-1	1	1	-1	-1	1	1	-1	1	-1	-1	-1	-1	-1	-1	1	1	1	-1
**26**	-1	1	-1	1	1	1	-1	1	1	-1	-1	-1	-1	-1	-1	1	1	1	-1	-1	1	1	1	1	-1	1	-1	1
**27**	1	1	-1	-1	1	1	1	1	1	1	-1	-1	-1	-1	1	1	-1	1	1	1	-1	1	1	1	1	-1	1	1

Supplementary Table 20: Weights of neuron 1 in 3-class binary 28×28 image classification.

**Index**	**0**	**1**	**2**	**3**	**4**	**5**	**6**	**7**	**8**	**9**	**10**	**11**	**12**	**13**	**14**	**15**	**16**	**17**	**18**	**19**	**20**	**21**	**22**	**23**	**24**	**25**	**26**	**27**
**0**	1	-1	1	1	1	-1	1	1	1	1	1	1	1	1	1	-1	1	-1	1	-1	1	-1	1	-1	1	-1	1	1
**1**	1	1	1	1	1	1	1	1	-1	-1	-1	1	1	1	-1	1	1	1	-1	1	1	-1	1	-1	-1	-1	-1	1
**2**	1	1	-1	1	1	-1	1	1	1	1	1	-1	1	1	1	-1	1	-1	-1	1	1	1	1	1	1	1	1	1
**3**	1	1	1	-1	1	-1	-1	1	-1	-1	1	-1	-1	-1	-1	-1	-1	-1	1	1	1	1	-1	1	-1	-1	1	-1
**4**	1	1	1	1	1	-1	1	-1	-1	-1	-1	-1	-1	-1	1	1	1	-1	-1	1	-1	-1	1	1	1	-1	1	-1
**5**	-1	1	1	-1	1	-1	1	1	-1	-1	-1	-1	-1	-1	-1	-1	-1	-1	1	1	1	1	1	1	1	1	1	-1
**6**	1	1	1	-1	1	-1	-1	-1	-1	-1	-1	-1	-1	-1	1	-1	-1	-1	1	1	1	1	1	1	-1	-1	-1	-1
**7**	-1	1	1	1	1	-1	-1	-1	-1	-1	-1	-1	1	-1	-1	-1	-1	-1	-1	1	-1	1	-1	1	1	1	1	-1
**8**	-1	1	1	-1	1	1	1	-1	-1	-1	-1	-1	1	1	-1	1	1	1	-1	-1	1	-1	1	-1	-1	-1	-1	1
**9**	-1	-1	1	1	-1	-1	-1	-1	-1	-1	1	-1	1	1	1	1	1	1	-1	-1	1	-1	1	-1	1	1	1	1
**10**	-1	-1	1	-1	-1	1	-1	1	-1	-1	-1	-1	-1	1	1	1	-1	-1	1	1	-1	-1	-1	-1	-1	1	1	1
**11**	1	1	1	1	-1	1	-1	-1	-1	-1	-1	-1	1	1	1	1	1	-1	-1	-1	-1	-1	-1	1	1	-1	1	1
**12**	1	-1	1	1	1	1	-1	-1	-1	1	1	-1	1	1	1	1	1	-1	-1	-1	1	1	-1	-1	-1	1	-1	1
**13**	-1	-1	-1	-1	1	1	-1	-1	-1	-1	-1	1	1	1	1	1	-1	-1	-1	-1	1	-1	-1	1	-1	-1	1	1
**14**	1	1	1	1	-1	-1	-1	-1	-1	-1	-1	-1	1	1	1	1	1	-1	-1	-1	-1	-1	-1	-1	-1	-1	-1	-1
**15**	1	1	-1	1	1	1	-1	-1	1	-1	-1	-1	1	1	1	1	-1	-1	-1	-1	-1	-1	-1	-1	-1	1	1	1
**16**	1	1	1	1	1	-1	-1	-1	-1	-1	-1	-1	-1	1	1	1	1	-1	-1	-1	-1	1	-1	-1	-1	-1	-1	1
**17**	1	1	-1	1	1	-1	-1	-1	-1	-1	-1	-1	-1	1	1	1	-1	-1	-1	-1	-1	-1	-1	-1	-1	-1	1	-1
**18**	1	1	1	-1	-1	1	-1	-1	-1	-1	-1	-1	-1	1	1	1	-1	-1	-1	-1	-1	-1	-1	-1	-1	-1	1	-1
**19**	-1	-1	1	1	-1	-1	-1	-1	-1	-1	-1	-1	-1	1	-1	-1	-1	1	-1	-1	1	-1	-1	-1	1	-1	1	-1
**20**	-1	1	-1	-1	-1	1	1	1	-1	-1	-1	-1	-1	-1	-1	1	1	-1	-1	-1	-1	-1	-1	1	-1	-1	1	1
**21**	-1	1	1	-1	1	1	1	-1	-1	-1	-1	1	-1	-1	-1	-1	-1	-1	-1	-1	-1	-1	1	-1	1	1	1	1
**22**	1	-1	1	-1	1	-1	-1	1	1	1	1	-1	-1	-1	1	-1	-1	1	1	-1	-1	-1	-1	1	1	-1	1	1
**23**	1	-1	1	1	1	1	1	1	1	-1	-1	1	1	1	1	-1	1	-1	-1	-1	1	-1	-1	1	1	-1	1	1
**24**	1	-1	1	-1	1	1	1	-1	-1	1	1	1	-1	1	-1	1	-1	1	-1	1	-1	-1	1	1	1	1	-1	1
**25**	1	-1	1	1	-1	-1	1	1	1	1	1	1	1	1	-1	1	-1	-1	-1	1	1	-1	1	1	-1	-1	-1	1
**26**	1	1	1	1	-1	1	1	-1	-1	1	1	1	1	1	-1	1	-1	1	-1	-1	-1	1	1	1	1	-1	1	1
**27**	1	1	-1	1	-1	-1	1	-1	-1	1	1	1	-1	1	1	1	-1	-1	-1	-1	-1	1	-1	1	1	1	-1	1

Supplementary Table 21: Weights of neuron 2 in 3-class binary 28 × 28 image classification.

**Index**	**0**	**1**	**2**	**3**	**4**	**5**	**6**	**7**	**8**	**9**	**10**	**11**	**12**	**13**	**14**	**15**	**16**	**17**	**18**	**19**	**20**	**21**	**22**	**23**	**24**	**25**	**26**	**27**
**0**	-1	1	-1	-1	1	1	-1	1	1	1	1	1	1	-1	1	1	1	-1	-1	-1	-1	1	1	-1	1	1	1	1
**1**	1	-1	-1	1	-1	-1	-1	1	-1	-1	-1	-1	-1	1	1	-1	1	1	-1	-1	-1	-1	-1	-1	1	-1	1	-1
**2**	1	1	-1	1	-1	-1	-1	-1	1	-1	-1	-1	1	-1	-1	-1	-1	-1	1	-1	1	-1	-1	-1	1	1	-1	1
**3**	-1	-1	-1	1	-1	1	1	-1	1	1	1	1	1	-1	1	1	-1	-1	1	-1	1	-1	-1	-1	1	1	-1	-1
**4**	1	1	-1	1	-1	-1	1	1	-1	1	1	-1	1	1	1	-1	1	-1	-1	1	-1	-1	-1	-1	1	-1	1	-1
**5**	1	-1	1	-1	1	-1	1	1	-1	-1	1	1	-1	1	1	1	1	-1	1	1	1	-1	1	-1	-1	-1	1	1
**6**	1	1	-1	-1	-1	-1	1	1	1	1	1	1	1	1	1	1	1	-1	1	1	1	-1	1	-1	-1	1	-1	1
**7**	1	1	-1	1	-1	-1	1	-1	1	1	-1	1	1	1	1	1	-1	1	-1	-1	-1	1	-1	1	-1	-1	1	1
**8**	-1	-1	1	1	-1	1	1	1	1	1	1	1	1	-1	1	-1	1	-1	-1	-1	-1	-1	1	-1	-1	-1	1	-1
**9**	1	-1	-1	1	1	1	1	-1	1	-1	-1	-1	1	-1	-1	-1	-1	-1	-1	1	1	-1	-1	1	1	1	1	1
**10**	1	1	-1	-1	1	-1	1	-1	1	-1	-1	-1	-1	-1	-1	-1	-1	1	1	-1	-1	-1	-1	-1	-1	1	-1	1
**11**	1	1	1	-1	1	-1	-1	-1	-1	-1	-1	-1	-1	-1	-1	-1	-1	1	-1	-1	-1	1	-1	-1	-1	-1	1	1
**12**	-1	1	1	1	-1	-1	-1	-1	-1	-1	-1	-1	-1	-1	-1	-1	-1	-1	1	1	1	-1	-1	-1	1	-1	-1	-1
**13**	1	1	-1	-1	-1	-1	-1	-1	-1	-1	-1	-1	-1	-1	1	-1	1	1	1	-1	-1	-1	-1	1	1	1	-1	1
**14**	-1	1	-1	1	-1	-1	-1	-1	-1	-1	-1	1	1	1	1	1	1	1	1	1	-1	-1	-1	-1	1	1	1	-1
**15**	1	-1	1	1	1	-1	-1	1	-1	1	1	1	1	1	1	1	1	1	1	1	1	-1	-1	-1	1	1	1	1
**16**	1	1	1	-1	1	-1	-1	-1	1	1	-1	1	1	1	1	1	1	1	1	1	1	1	-1	1	-1	1	-1	-1
**17**	-1	-1	1	-1	-1	1	1	1	1	1	1	1	1	1	1	1	1	1	-1	1	-1	1	1	1	1	1	1	1
**18**	-1	1	1	1	1	1	1	1	1	1	1	1	1	1	1	-1	1	1	1	-1	1	1	1	1	-1	1	-1	1
**19**	1	1	-1	1	-1	1	1	1	1	-1	1	1	1	-1	-1	1	1	1	-1	1	1	1	1	1	-1	1	1	1
**20**	1	1	1	-1	1	1	1	1	1	-1	1	-1	-1	-1	1	1	-1	-1	1	1	1	1	1	1	1	-1	-1	1
**21**	-1	1	-1	1	1	1	1	-1	1	1	-1	-1	-1	1	-1	1	-1	-1	1	-1	1	1	1	1	-1	1	1	-1
**22**	1	-1	-1	1	1	-1	1	1	-1	-1	1	-1	-1	-1	-1	1	1	-1	1	-1	1	1	1	1	-1	-1	-1	1
**23**	-1	-1	-1	1	1	-1	-1	-1	-1	1	1	1	-1	-1	-1	-1	-1	-1	1	1	1	1	-1	-1	1	-1	-1	1
**24**	1	1	-1	1	-1	-1	1	1	-1	1	-1	1	-1	-1	1	1	1	1	1	-1	-1	-1	1	1	1	1	1	1
**25**	1	-1	1	-1	-1	-1	1	-1	-1	1	-1	1	1	1	1	1	-1	1	1	-1	-1	-1	-1	-1	1	1	1	1
**26**	-1	1	1	-1	-1	1	-1	1	1	-1	1	-1	1	1	1	-1	-1	-1	-1	-1	1	-1	-1	-1	1	1	-1	1
**27**	1	-1	1	-1	-1	-1	-1	1	-1	1	-1	-1	-1	1	-1	1	1	-1	-1	1	1	-1	1	1	-1	-1	1	1

Supplementary Table 22: Weights of neuron 0 in 2-class 3-bit 8×8 image classification.

**Index**	**0**	**1**	**2**	**3**	**4**	**5**	**6**	**7**
**0**	-1	-1	-1	1	-1	1	1	-1
**1**	-1	1	1	1	-1	1	-1	-1
**2**	-1	-1	1	1	-1	1	1	1
**3**	-1	-1	1	1	-1	1	1	-1
**4**	1	1	1	-1	-1	1	1	1
**5**	-1	1	1	-1	-1	1	1	1
**6**	1	-1	1	1	1	-1	-1	1
**7**	-1	-1	-1	1	1	-1	-1	-1

Supplementary Table 23: Weights of neuron 1 in 2-class 3-bit 8×8 image classification.

**Index**	**0**	**1**	**2**	**3**	**4**	**5**	**6**	**7**
**0**	1	-1	1	1	1	-1	-1	1
**1**	1	1	-1	-1	-1	1	1	-1
**2**	1	1	-1	-1	-1	-1	-1	1
**3**	1	-1	-1	-1	1	-1	-1	1
**4**	-1	-1	-1	1	1	-1	-1	1
**5**	-1	-1	-1	-1	-1	-1	-1	1
**6**	1	-1	-1	-1	1	-1	-1	1
**7**	1	1	1	1	-1	1	-1	-1

Supplementary Table 24: Weights of neuron 0 in 2-class 3-bit 12×12 image classification.

**Index**	**0**	**1**	**2**	**3**	**4**	**5**	**6**	**7**	**8**	**9**	**10**	**11**
**0**	1	-1	-1	1	-1	1	-1	1	-1	-1	-1	-1
**1**	1	-1	1	-1	-1	1	-1	-1	1	-1	-1	1
**2**	-1	-1	-1	1	1	1	1	1	-1	1	-1	-1
**3**	-1	-1	1	-1	1	1	1	1	1	1	-1	1
**4**	1	-1	1	-1	-1	1	-1	-1	1	1	1	-1
**5**	1	1	-1	1	1	-1	-1	-1	1	1	-1	1
**6**	-1	-1	1	1	1	-1	-1	1	1	1	1	1
**7**	-1	1	1	1	1	-1	-1	1	1	1	1	1
**8**	1	-1	1	1	1	-1	-1	1	-1	-1	-1	1
**9**	1	1	-1	1	1	1	1	-1	-1	1	-1	-1
**10**	1	-1	1	-1	1	-1	1	-1	-1	-1	-1	-1
**11**	1	-1	1	1	-1	-1	-1	-1	1	-1	-1	-1

Supplementary Table 25: Weights of neuron 1 in 2-class 3-bit 12×12 image classification.

**Index**	**0**	**1**	**2**	**3**	**4**	**5**	**6**	**7**	**8**	**9**	**10**	**11**
**0**	1	-1	1	1	1	-1	-1	1	1	1	1	-1
**1**	-1	-1	-1	1	-1	1	1	-1	1	-1	-1	1
**2**	1	1	1	1	-1	-1	1	-1	1	1	1	-1
**3**	-1	1	-1	1	-1	-1	1	-1	-1	-1	1	1
**4**	1	1	1	-1	-1	-1	1	1	-1	-1	-1	1
**5**	-1	-1	-1	-1	-1	-1	1	-1	-1	-1	1	1
**6**	-1	-1	-1	-1	-1	1	1	-1	-1	-1	-1	-1
**7**	-1	1	-1	-1	-1	1	1	-1	-1	1	1	1
**8**	-1	-1	-1	-1	-1	1	-1	1	-1	-1	-1	1
**9**	1	1	1	1	-1	-1	-1	-1	1	-1	-1	-1
**10**	1	1	1	-1	1	-1	1	1	-1	1	1	1
**11**	1	1	1	-1	1	1	1	1	-1	-1	1	-1

Supplementary Table 26: Weights of neuron 0 in 2-class 3-bit 16×16 image classification.

**Index**	**0**	**1**	**2**	**3**	**4**	**5**	**6**	**7**	**8**	**9**	**10**	**11**	**12**	**13**	**14**	**15**
**0**	-1	1	-1	1	-1	-1	1	-1	1	-1	1	-1	-1	-1	1	1
**1**	-1	1	-1	-1	1	-1	-1	-1	-1	1	-1	1	1	1	-1	-1
**2**	-1	1	1	-1	1	1	-1	-1	-1	-1	1	-1	-1	1	1	-1
**3**	-1	-1	1	1	-1	1	1	1	-1	1	1	-1	1	-1	-1	1
**4**	-1	-1	1	1	-1	-1	1	1	1	1	-1	1	1	1	1	-1
**5**	-1	1	-1	1	1	1	1	-1	-1	-1	1	1	1	1	1	1
**6**	-1	-1	1	1	-1	1	1	-1	-1	-1	1	-1	1	1	1	1
**7**	-1	-1	-1	-1	1	1	1	-1	-1	-1	1	1	1	1	1	1
**8**	-1	-1	1	1	1	1	1	-1	-1	-1	1	1	1	1	1	-1
**9**	-1	1	-1	-1	1	1	-1	-1	-1	1	1	1	1	1	-1	1
**10**	-1	-1	1	1	1	1	1	-1	-1	-1	1	1	1	-1	1	-1
**11**	1	1	1	1	-1	1	1	-1	1	-1	-1	1	1	1	1	-1
**12**	-1	-1	-1	-1	1	-1	1	1	-1	-1	-1	-1	-1	-1	-1	-1
**13**	1	-1	-1	1	-1	1	1	1	-1	1	1	-1	-1	1	1	-1
**14**	-1	1	-1	-1	-1	-1	1	-1	1	-1	1	1	-1	-1	1	1
**15**	1	-1	-1	-1	1	-1	1	-1	-1	-1	-1	-1	1	1	-1	-1

Supplementary Table 27: Weights of neuron 1 in 2-class 3-bit 16×16 image classification.

**Index**	**0**	**1**	**2**	**3**	**4**	**5**	**6**	**7**	**8**	**9**	**10**	**11**	**12**	**13**	**14**	**15**
**0**	-1	1	1	-1	1	1	-1	1	1	1	1	-1	1	1	-1	1
**1**	1	1	-1	1	1	1	1	1	1	1	-1	-1	-1	1	1	1
**2**	1	1	1	-1	1	1	-1	-1	1	1	-1	1	-1	1	1	-1
**3**	1	-1	1	1	-1	1	1	-1	1	-1	-1	1	1	1	1	-1
**4**	1	1	1	-1	1	-1	-1	1	-1	-1	1	-1	-1	-1	-1	1
**5**	-1	-1	-1	1	-1	-1	-1	-1	1	1	-1	1	-1	1	-1	1
**6**	1	-1	1	-1	-1	-1	-1	-1	1	1	-1	-1	-1	-1	1	-1
**7**	-1	-1	-1	-1	-1	-1	-1	1	1	1	-1	-1	-1	-1	1	-1
**8**	1	1	1	-1	1	-1	-1	1	1	1	-1	-1	-1	-1	1	1
**9**	-1	1	1	-1	-1	-1	-1	1	1	-1	-1	-1	-1	1	1	-1
**10**	-1	-1	-1	-1	-1	-1	-1	1	1	-1	-1	-1	-1	-1	1	-1
**11**	-1	-1	-1	-1	-1	-1	-1	1	-1	1	1	-1	-1	-1	1	1
**12**	-1	1	1	1	-1	1	-1	-1	-1	1	1	-1	1	1	1	-1
**13**	1	1	1	-1	1	-1	-1	-1	-1	-1	1	-1	1	-1	-1	1
**14**	1	-1	1	-1	-1	1	1	-1	-1	-1	-1	1	1	1	1	1
**15**	-1	-1	-1	1	-1	-1	1	-1	-1	-1	1	-1	1	1	1	-1

Supplementary Table 28: Weights of neuron 0 in 2-class 3-bit 28×28 image classification.

**Index**	**0**	**1**	**2**	**3**	**4**	**5**	**6**	**7**	**8**	**9**	**10**	**11**	**12**	**13**	**14**	**15**	**16**	**17**	**18**	**19**	**20**	**21**	**22**	**23**	**24**	**25**	**26**	**27**
**0**	1	-1	1	1	1	-1	1	-1	1	1	1	-1	-1	-1	-1	-1	-1	1	1	-1	1	-1	-1	1	1	-1	-1	1
**1**	-1	-1	1	1	1	-1	-1	-1	1	1	-1	-1	-1	1	1	-1	-1	1	1	1	-1	1	1	1	1	-1	-1	-1
**2**	-1	-1	-1	-1	-1	-1	1	1	-1	1	-1	1	-1	-1	-1	-1	-1	1	1	-1	1	1	-1	-1	-1	1	1	1
**3**	-1	-1	-1	-1	1	1	-1	1	-1	1	-1	1	1	-1	1	-1	1	1	-1	-1	1	-1	1	-1	-1	-1	1	1
**4**	1	-1	-1	1	1	-1	-1	-1	-1	-1	1	1	1	1	-1	-1	-1	1	1	1	1	-1	1	1	1	-1	-1	-1
**5**	1	-1	-1	-1	1	-1	1	-1	-1	-1	-1	-1	1	1	-1	1	-1	-1	-1	-1	-1	1	-1	-1	1	-1	-1	1
**6**	-1	1	-1	1	1	1	-1	-1	1	1	1	-1	1	-1	1	1	1	1	1	1	-1	1	-1	-1	-1	-1	1	1
**7**	-1	-1	1	1	1	1	-1	-1	1	-1	1	1	1	1	1	1	1	1	1	-1	-1	-1	1	1	-1	1	1	1
**8**	1	-1	-1	-1	-1	1	1	-1	-1	1	1	1	-1	-1	1	-1	-1	1	-1	1	1	1	1	1	-1	-1	1	1
**9**	-1	-1	1	1	-1	-1	-1	1	1	1	-1	1	1	1	-1	-1	1	1	1	1	-1	-1	1	-1	-1	-1	1	1
**10**	1	1	1	-1	-1	-1	-1	1	-1	-1	-1	1	1	1	1	-1	-1	1	-1	1	1	-1	1	1	-1	1	-1	1
**11**	-1	1	-1	1	-1	-1	1	-1	1	1	1	-1	1	-1	1	-1	-1	-1	-1	1	1	1	1	-1	1	-1	-1	1
**12**	-1	-1	1	1	-1	-1	1	1	1	1	1	1	-1	-1	-1	-1	-1	1	1	1	1	1	1	1	-1	1	-1	1
**13**	-1	-1	-1	1	1	1	1	-1	1	1	1	-1	1	-1	-1	-1	-1	-1	1	1	1	1	-1	1	-1	-1	-1	-1
**14**	-1	-1	1	-1	-1	1	1	1	1	1	1	1	-1	-1	-1	-1	-1	-1	1	1	1	1	1	1	-1	-1	-1	1
**15**	1	-1	1	1	-1	1	1	-1	1	1	1	-1	-1	-1	-1	-1	-1	1	1	1	1	1	-1	1	1	-1	1	-1
**16**	-1	1	1	-1	1	-1	1	1	1	-1	1	1	1	-1	-1	-1	1	-1	1	-1	1	1	1	-1	1	-1	-1	-1
**17**	1	1	1	-1	1	1	1	1	1	1	-1	1	1	-1	-1	1	-1	1	1	-1	1	1	-1	1	1	1	1	-1
**18**	1	-1	1	-1	-1	1	1	1	1	1	1	-1	-1	1	-1	1	1	1	1	-1	-1	-1	1	1	-1	1	1	-1
**19**	-1	1	1	1	-1	-1	1	1	-1	1	-1	1	1	-1	1	-1	1	-1	1	1	-1	1	1	-1	-1	-1	-1	-1
**20**	-1	1	1	-1	-1	-1	1	1	1	-1	1	1	-1	1	-1	1	-1	1	-1	1	-1	-1	1	1	1	1	-1	1
**21**	1	1	-1	1	-1	1	-1	1	1	1	-1	1	1	-1	-1	-1	-1	-1	-1	-1	-1	-1	-1	-1	-1	-1	-1	-1
**22**	1	1	1	1	1	1	1	-1	1	1	1	1	1	1	1	-1	1	1	-1	-1	-1	1	-1	-1	-1	1	-1	1
**23**	-1	-1	-1	-1	-1	-1	1	-1	-1	-1	1	-1	1	1	1	1	1	-1	-1	-1	1	1	-1	-1	-1	-1	1	-1
**24**	-1	-1	1	1	1	-1	-1	1	1	-1	1	-1	-1	1	1	1	-1	-1	-1	-1	-1	1	-1	-1	1	-1	1	-1
**25**	-1	1	-1	-1	-1	-1	1	-1	-1	-1	-1	-1	1	1	-1	1	-1	1	1	-1	-1	-1	1	1	1	-1	1	1
**26**	1	1	-1	1	1	1	-1	-1	1	1	-1	1	-1	1	1	-1	-1	1	-1	-1	-1	1	-1	1	1	1	-1	1
**27**	1	-1	1	-1	1	-1	1	-1	-1	-1	1	-1	-1	-1	-1	1	-1	-1	1	1	1	1	1	1	-1	-1	1	-1

Supplementary Table 29: Weights of neuron 1 in 2-class 3-bit 28×28 image classification.

**Index**	**0**	**1**	**2**	**3**	**4**	**5**	**6**	**7**	**8**	**9**	**10**	**11**	**12**	**13**	**14**	**15**	**16**	**17**	**18**	**19**	**20**	**21**	**22**	**23**	**24**	**25**	**26**	**27**
**0**	-1	-1	1	1	1	-1	-1	-1	-1	-1	-1	-1	-1	1	1	1	1	-1	-1	1	1	-1	1	-1	1	-1	1	1
**1**	1	1	1	1	1	-1	1	1	1	1	1	-1	-1	-1	-1	-1	-1	-1	1	1	1	-1	-1	-1	1	-1	1	-1
**2**	1	-1	1	-1	1	-1	1	-1	-1	-1	-1	1	-1	1	-1	1	1	-1	1	1	1	1	1	-1	-1	1	-1	1
**3**	-1	-1	1	-1	-1	1	1	1	-1	1	1	-1	-1	-1	1	-1	-1	1	-1	1	1	1	1	1	-1	1	-1	1
**4**	-1	1	1	1	1	1	-1	-1	1	-1	1	1	1	-1	1	1	1	1	-1	1	1	-1	1	-1	-1	1	1	1
**5**	1	1	-1	1	-1	1	-1	1	-1	1	1	1	1	-1	-1	-1	-1	-1	-1	-1	1	1	-1	1	1	1	-1	-1
**6**	1	1	1	1	1	-1	1	1	1	-1	-1	-1	1	-1	-1	1	-1	1	1	1	-1	1	-1	1	1	-1	1	-1
**7**	-1	-1	1	-1	-1	-1	-1	-1	1	1	-1	-1	-1	-1	-1	1	-1	-1	-1	-1	1	-1	-1	1	-1	-1	-1	1
**8**	-1	1	1	-1	-1	-1	-1	1	-1	-1	1	-1	1	1	1	-1	-1	1	-1	-1	1	-1	-1	1	1	1	-1	1
**9**	-1	1	-1	-1	-1	1	1	-1	-1	1	1	1	-1	-1	1	-1	-1	-1	-1	1	-1	-1	-1	-1	-1	-1	1	1
**10**	1	1	1	1	-1	1	1	-1	-1	1	1	1	-1	-1	-1	1	1	-1	-1	-1	-1	-1	1	-1	-1	-1	1	-1
**11**	-1	1	1	-1	1	-1	1	-1	1	-1	-1	-1	-1	-1	-1	1	1	-1	-1	-1	-1	-1	-1	-1	-1	-1	1	-1
**12**	1	-1	-1	1	-1	-1	1	-1	-1	-1	-1	-1	1	1	1	1	1	1	-1	-1	-1	1	1	1	1	-1	-1	-1
**13**	1	-1	1	1	1	-1	-1	-1	-1	-1	1	-1	-1	1	1	1	1	1	-1	-1	1	-1	-1	-1	1	-1	1	1
**14**	1	1	-1	-1	-1	-1	-1	-1	1	-1	1	-1	1	1	1	1	-1	-1	1	-1	-1	1	-1	-1	-1	1	-1	-1
**15**	1	1	1	1	-1	1	-1	-1	-1	-1	-1	-1	1	1	1	1	1	-1	1	-1	-1	-1	-1	-1	-1	-1	1	1
**16**	1	1	-1	-1	1	-1	-1	-1	1	-1	-1	-1	-1	1	1	1	1	-1	-1	-1	1	-1	-1	1	-1	-1	1	1
**17**	1	1	-1	-1	-1	-1	1	-1	-1	-1	-1	1	1	1	1	-1	-1	1	-1	-1	-1	-1	-1	1	1	1	1	-1
**18**	1	-1	-1	1	1	1	-1	-1	-1	-1	-1	1	-1	1	1	-1	-1	-1	-1	1	1	-1	1	-1	1	-1	1	-1
**19**	1	-1	-1	1	1	-1	-1	-1	-1	-1	-1	1	1	-1	-1	-1	-1	-1	-1	-1	1	1	1	-1	1	1	1	-1
**20**	-1	1	1	1	-1	-1	1	-1	1	1	-1	1	-1	1	1	1	1	-1	1	-1	1	-1	-1	1	1	-1	1	-1
**21**	-1	1	1	1	1	-1	1	1	1	1	-1	-1	1	-1	-1	-1	-1	1	1	1	1	1	1	1	1	-1	1	-1
**22**	-1	-1	-1	1	1	1	1	1	-1	-1	-1	-1	-1	-1	1	1	1	-1	-1	-1	1	1	1	-1	-1	-1	-1	-1
**23**	-1	-1	-1	1	1	-1	-1	1	1	-1	1	-1	1	-1	-1	1	1	1	-1	-1	-1	-1	-1	-1	-1	-1	-1	-1
**24**	-1	1	-1	-1	1	-1	1	1	-1	1	-1	-1	-1	1	-1	1	1	-1	-1	1	1	1	1	1	-1	-1	1	-1
**25**	-1	1	-1	1	-1	1	-1	-1	-1	1	1	-1	1	1	1	-1	-1	1	1	-1	1	1	1	1	1	1	1	1
**26**	1	-1	-1	1	1	-1	1	1	-1	1	1	1	1	1	1	1	-1	1	-1	-1	-1	-1	1	-1	1	1	1	-1
**27**	-1	1	1	1	1	1	-1	1	1	1	1	-1	1	1	1	1	-1	1	-1	-1	1	1	-1	1	-1	1	1	-1

Supplementary Table 30: Weights of neuron 0 in 3-class 3-bit 8×8 image classification.

**Index**	**0**	**1**	**2**	**3**	**4**	**5**	**6**	**7**
**0**	-1	1	1	-1	-1	-1	-1	-1
**1**	-1	-1	-1	-1	1	1	1	-1
**2**	-1	-1	1	-1	1	1	1	1
**3**	1	1	1	1	-1	1	1	1
**4**	-1	1	1	-1	-1	1	1	-1
**5**	-1	-1	1	-1	-1	1	1	-1
**6**	-1	-1	-1	1	1	1	-1	-1
**7**	1	1	-1	-1	-1	-1	-1	-1

Supplementary Table 31: Weights of neuron 1 in 3-class 3-bit 8×8 image classification.

**Index**	**0**	**1**	**2**	**3**	**4**	**5**	**6**	**7**
**0**	1	-1	-1	-1	-1	-1	-1	-1
**1**	-1	-1	-1	-1	-1	1	1	-1
**2**	-1	-1	-1	-1	1	-1	-1	-1
**3**	-1	-1	-1	-1	1	-1	-1	-1
**4**	-1	-1	-1	1	1	-1	-1	-1
**5**	-1	-1	-1	1	-1	-1	-1	-1
**6**	-1	-1	-1	-1	-1	-1	-1	-1
**7**	-1	1	1	-1	-1	-1	-1	-1

Supplementary Table 32: Weights of neuron 2 in 3-class 3-bit 8×8 image classification.

**Index**	**0**	**1**	**2**	**3**	**4**	**5**	**6**	**7**
**0**	-1	-1	1	1	1	1	1	1
**1**	-1	1	1	1	1	-1	-1	1
**2**	1	1	1	-1	-1	-1	1	1
**3**	-1	-1	-1	-1	-1	1	-1	1
**4**	1	-1	-1	1	1	1	-1	1
**5**	1	1	1	1	-1	1	1	1
**6**	1	1	1	-1	-1	1	1	1
**7**	1	-1	-1	1	1	1	1	1

Supplementary Table 33: Weights of neuron 0 in 3-class 3-bit 12×12 image classification.

**Index**	**0**	**1**	**2**	**3**	**4**	**5**	**6**	**7**	**8**	**9**	**10**	**11**
**0**	1	-1	1	1	1	1	-1	1	1	-1	-1	-1
**1**	1	1	-1	1	-1	1	-1	1	1	1	1	-1
**2**	1	-1	-1	-1	-1	-1	1	1	1	-1	1	1
**3**	1	-1	1	1	-1	-1	1	1	1	1	1	-1
**4**	1	-1	1	1	1	1	1	1	-1	1	1	-1
**5**	1	-1	1	1	1	1	-1	-1	1	1	1	-1
**6**	1	-1	1	1	1	-1	-1	-1	1	1	1	-1
**7**	-1	-1	-1	1	1	-1	-1	1	1	1	-1	-1
**8**	1	-1	-1	1	1	-1	1	1	-1	-1	-1	-1
**9**	-1	-1	-1	-1	1	1	1	1	-1	-1	-1	-1
**10**	-1	1	-1	-1	-1	1	1	1	-1	-1	-1	-1
**11**	-1	1	-1	-1	1	-1	-1	1	1	1	-1	-1

Supplementary Table 34: Weights of neuron 1 in 3-class 3-bit 12×12 image classification.

**Index**	**0**	**1**	**2**	**3**	**4**	**5**	**6**	**7**	**8**	**9**	**10**	**11**
**0**	1	-1	1	-1	-1	-1	1	-1	1	1	-1	1
**1**	1	1	1	-1	-1	-1	1	-1	-1	-1	-1	1
**2**	1	1	-1	-1	-1	-1	-1	-1	1	1	1	-1
**3**	-1	-1	-1	-1	-1	-1	1	1	-1	-1	-1	-1
**4**	1	-1	-1	-1	-1	-1	1	1	-1	-1	-1	-1
**5**	-1	-1	1	1	-1	1	1	-1	-1	-1	-1	-1
**6**	-1	-1	-1	-1	-1	1	1	-1	-1	-1	-1	-1
**7**	1	-1	-1	-1	-1	1	1	-1	-1	-1	-1	-1
**8**	-1	-1	-1	-1	-1	1	-1	-1	-1	-1	-1	-1
**9**	1	-1	-1	1	1	-1	-1	1	-1	-1	1	-1
**10**	-1	1	1	1	-1	-1	-1	-1	-1	-1	-1	-1
**11**	-1	1	-1	-1	1	1	1	1	-1	1	-1	-1

Supplementary Table 35: Weights of neuron 2 in 3-class 3-bit 12×12 image classification.

**Index**	**0**	**1**	**2**	**3**	**4**	**5**	**6**	**7**	**8**	**9**	**10**	**11**
**0**	1	-1	-1	1	-1	1	-1	1	-1	-1	1	-1
**1**	1	-1	1	1	1	1	1	1	-1	1	1	-1
**2**	-1	1	-1	1	1	1	1	1	-1	-1	-1	1
**3**	1	1	1	1	1	1	-1	-1	-1	-1	-1	-1
**4**	-1	1	1	-1	-1	-1	-1	1	-1	-1	-1	1
**5**	-1	-1	-1	-1	-1	-1	-1	1	-1	-1	-1	1
**6**	-1	1	-1	-1	1	1	1	1	1	-1	1	1
**7**	-1	1	1	1	1	1	1	1	1	1	1	1
**8**	-1	1	1	1	-1	1	-1	1	1	1	1	1
**9**	-1	1	1	1	-1	-1	-1	1	1	1	1	1
**10**	-1	-1	-1	-1	1	-1	-1	1	1	1	1	1
**11**	1	-1	1	1	-1	1	1	-1	-1	-1	-1	-1

Supplementary Table 36: Weights of neuron 0 in 3-class 3-bit 16×16 image classification.

**Index**	**0**	**1**	**2**	**3**	**4**	**5**	**6**	**7**	**8**	**9**	**10**	**11**	**12**	**13**	**14**	**15**
**0**	1	-1	1	-1	-1	1	1	1	1	1	1	1	-1	-1	-1	1
**1**	1	1	1	1	1	-1	1	-1	-1	1	-1	-1	1	-1	-1	1
**2**	-1	-1	1	-1	-1	1	-1	1	1	-1	1	1	1	1	1	1
**3**	-1	-1	-1	1	-1	1	-1	-1	1	-1	-1	-1	-1	1	1	1
**4**	1	-1	-1	-1	-1	-1	-1	1	-1	1	1	-1	-1	1	1	1
**5**	-1	-1	-1	-1	1	-1	-1	1	1	-1	1	-1	1	1	-1	-1
**6**	1	-1	-1	1	1	1	1	1	1	-1	1	-1	1	1	1	-1
**7**	1	-1	1	1	1	1	1	-1	-1	-1	1	1	1	1	1	-1
**8**	1	-1	1	1	1	1	1	-1	-1	-1	-1	1	1	1	-1	-1
**9**	1	-1	1	1	1	1	-1	-1	-1	-1	1	-1	1	1	-1	-1
**10**	1	-1	-1	-1	1	1	-1	-1	-1	-1	1	-1	1	1	-1	-1
**11**	1	-1	-1	-1	1	1	1	-1	-1	-1	1	-1	-1	-1	-1	-1
**12**	-1	-1	-1	1	-1	-1	1	1	1	-1	-1	-1	1	-1	-1	-1
**13**	-1	-1	-1	-1	-1	-1	1	1	1	1	-1	-1	-1	-1	-1	-1
**14**	-1	1	-1	-1	1	-1	1	-1	1	-1	-1	-1	-1	-1	-1	-1
**15**	1	1	-1	1	1	-1	-1	1	-1	-1	1	1	-1	1	1	1

Supplementary Table 37: Weights of neuron 1 in 3-class 3-bit 16×16 image classification.

**Index**	**0**	**1**	**2**	**3**	**4**	**5**	**6**	**7**	**8**	**9**	**10**	**11**	**12**	**13**	**14**	**15**
**0**	1	-1	1	1	-1	-1	-1	-1	-1	-1	-1	-1	1	1	1	-1
**1**	1	1	1	-1	1	1	-1	-1	1	1	-1	-1	-1	-1	-1	1
**2**	-1	1	1	1	-1	-1	-1	-1	-1	-1	-1	1	1	1	1	1
**3**	-1	-1	1	-1	-1	-1	-1	-1	-1	-1	-1	1	1	1	1	-1
**4**	-1	-1	1	-1	-1	-1	1	1	-1	1	-1	-1	1	-1	-1	-1
**5**	1	-1	-1	-1	-1	-1	-1	1	1	1	-1	1	-1	-1	1	-1
**6**	-1	-1	-1	-1	1	1	-1	1	1	1	-1	-1	-1	1	-1	-1
**7**	-1	1	-1	1	1	-1	-1	1	1	1	-1	1	1	-1	-1	-1
**8**	-1	-1	-1	1	-1	-1	-1	1	1	-1	-1	-1	-1	1	-1	-1
**9**	1	-1	-1	-1	1	-1	-1	1	1	-1	-1	-1	1	-1	-1	-1
**10**	1	-1	-1	-1	-1	-1	-1	-1	1	-1	-1	-1	-1	-1	-1	-1
**11**	-1	-1	-1	-1	-1	-1	-1	1	1	-1	1	-1	-1	-1	-1	-1
**12**	1	1	-1	-1	1	1	-1	-1	-1	-1	-1	-1	-1	1	1	-1
**13**	1	-1	-1	1	-1	-1	-1	1	1	1	-1	-1	-1	-1	1	1
**14**	1	1	1	1	1	1	-1	-1	-1	-1	-1	-1	-1	-1	-1	-1
**15**	1	-1	1	-1	1	1	1	1	1	1	1	-1	1	-1	-1	-1

Supplementary Table 38: Weights of neuron 2 in 3-class 3-bit 16×16 image classification.

**Index**	**0**	**1**	**2**	**3**	**4**	**5**	**6**	**7**	**8**	**9**	**10**	**11**	**12**	**13**	**14**	**15**
**0**	1	-1	-1	-1	-1	1	1	-1	1	-1	1	1	1	-1	1	1
**1**	1	-1	-1	1	1	1	1	1	1	1	1	1	1	1	1	-1
**2**	-1	-1	-1	1	1	1	1	1	1	1	1	-1	-1	-1	-1	-1
**3**	-1	-1	1	-1	1	1	1	-1	1	1	-1	-1	-1	-1	-1	-1
**4**	1	1	1	1	1	1	1	1	-1	-1	-1	-1	1	-1	-1	-1
**5**	-1	1	1	1	-1	1	1	-1	-1	-1	1	-1	1	1	-1	1
**6**	-1	1	1	-1	-1	-1	-1	-1	-1	1	1	-1	-1	-1	1	1
**7**	-1	1	-1	-1	-1	-1	-1	-1	-1	1	1	1	-1	-1	1	1
**8**	1	1	-1	-1	-1	-1	1	1	1	1	1	1	-1	-1	1	1
**9**	-1	1	1	1	1	1	1	1	1	1	1	1	1	1	1	1
**10**	-1	1	1	1	1	1	1	1	1	1	1	1	1	1	1	1
**11**	-1	1	1	1	1	1	1	-1	1	-1	1	1	1	1	1	1
**12**	-1	1	1	-1	1	1	-1	-1	-1	1	1	1	1	1	1	1
**13**	-1	1	1	1	1	-1	1	-1	-1	-1	1	1	1	1	1	1
**14**	1	-1	-1	1	1	1	-1	1	1	1	1	1	1	1	1	1
**15**	-1	1	-1	-1	-1	-1	-1	1	1	-1	1	1	-1	-1	-1	1

Supplementary Table 39: Weights of neuron 0 in 3-class 3-bit 28×28 image classification.

**Index**	**0**	**1**	**2**	**3**	**4**	**5**	**6**	**7**	**8**	**9**	**10**	**11**	**12**	**13**	**14**	**15**	**16**	**17**	**18**	**19**	**20**	**21**	**22**	**23**	**24**	**25**	**26**	**27**
**0**	1	-1	1	-1	1	-1	1	1	-1	1	1	-1	1	-1	1	1	-1	1	-1	-1	1	-1	-1	1	-1	1	1	1
**1**	-1	-1	-1	1	1	-1	1	-1	1	-1	1	1	1	1	-1	-1	1	1	-1	1	1	-1	1	1	1	-1	1	-1
**2**	-1	1	1	1	1	1	1	1	-1	-1	-1	1	-1	-1	-1	-1	-1	-1	-1	-1	-1	-1	1	1	-1	-1	-1	-1
**3**	1	-1	1	1	1	-1	-1	1	1	-1	-1	1	1	-1	-1	-1	-1	1	-1	1	-1	1	-1	1	1	1	1	1
**4**	1	-1	1	-1	1	-1	1	-1	1	-1	1	1	-1	-1	1	1	1	-1	-1	1	-1	1	1	1	1	-1	1	-1
**5**	1	-1	-1	-1	-1	-1	1	-1	-1	1	-1	-1	1	1	-1	1	-1	1	1	1	-1	-1	1	-1	1	-1	-1	1
**6**	1	1	-1	-1	-1	-1	-1	1	1	-1	1	1	-1	1	-1	-1	1	1	-1	-1	1	1	-1	1	1	1	1	1
**7**	1	1	-1	-1	-1	1	1	1	-1	1	1	1	-1	1	1	1	-1	1	1	1	-1	1	-1	1	1	-1	-1	1
**8**	1	-1	-1	-1	-1	-1	1	-1	-1	1	-1	-1	-1	1	-1	1	1	1	1	1	-1	1	-1	1	1	1	-1	1
**9**	-1	1	1	-1	-1	-1	1	1	1	1	-1	1	1	1	1	-1	1	-1	-1	-1	-1	-1	-1	-1	1	1	-1	1
**10**	-1	1	1	-1	-1	-1	1	-1	1	-1	-1	1	1	1	1	1	1	1	1	1	1	1	1	1	1	1	-1	-1
**11**	-1	1	1	-1	-1	1	1	1	1	1	1	1	1	1	-1	-1	-1	1	1	-1	-1	1	1	1	-1	-1	-1	-1
**12**	-1	1	1	-1	1	1	1	1	1	1	1	1	1	1	-1	-1	-1	1	-1	1	1	-1	1	1	1	-1	-1	-1
**13**	1	1	1	-1	1	1	1	1	1	1	1	1	-1	-1	-1	-1	-1	1	1	1	1	1	1	1	1	-1	-1	-1
**14**	-1	1	1	-1	1	1	1	1	1	-1	1	1	-1	-1	-1	-1	-1	-1	1	-1	1	1	-1	1	-1	-1	-1	-1
**15**	-1	-1	1	-1	-1	1	-1	1	1	1	1	-1	-1	-1	-1	-1	-1	-1	-1	1	-1	1	1	1	-1	-1	-1	-1
**16**	1	-1	-1	-1	1	1	1	1	1	1	1	-1	-1	-1	-1	-1	-1	-1	-1	1	-1	-1	1	1	1	-1	-1	-1
**17**	1	1	-1	-1	-1	-1	1	1	-1	1	1	-1	-1	-1	-1	-1	-1	1	1	-1	1	-1	-1	1	-1	-1	-1	-1
**18**	1	1	-1	-1	1	-1	-1	-1	1	1	1	-1	-1	-1	-1	-1	-1	-1	1	-1	1	-1	1	-1	-1	-1	-1	-1
**19**	1	1	1	-1	-1	1	-1	-1	-1	1	1	-1	1	-1	-1	1	1	1	-1	-1	-1	1	-1	1	-1	-1	-1	-1
**20**	1	1	-1	-1	-1	-1	1	-1	1	-1	-1	1	-1	-1	-1	-1	-1	1	1	-1	-1	-1	-1	-1	1	-1	-1	1
**21**	1	1	-1	-1	1	1	-1	1	-1	1	-1	1	1	1	-1	1	-1	-1	-1	-1	1	1	1	-1	-1	-1	-1	-1
**22**	1	1	-1	1	-1	1	-1	-1	-1	1	-1	1	1	1	1	1	-1	-1	-1	-1	-1	1	-1	-1	1	-1	1	-1
**23**	1	-1	-1	-1	-1	-1	-1	1	-1	-1	1	-1	1	1	1	1	1	1	-1	-1	-1	-1	-1	-1	-1	-1	-1	1
**24**	1	1	-1	1	-1	-1	-1	-1	-1	-1	-1	1	-1	1	1	1	-1	-1	-1	-1	-1	-1	-1	-1	-1	-1	1	1
**25**	-1	1	1	-1	-1	-1	1	-1	-1	-1	1	1	-1	-1	-1	-1	-1	-1	-1	-1	-1	1	1	1	-1	1	-1	-1
**26**	1	-1	-1	1	-1	1	1	-1	-1	-1	-1	-1	-1	-1	-1	-1	-1	1	-1	-1	-1	-1	-1	-1	-1	1	-1	1
**27**	-1	-1	-1	-1	-1	1	1	1	1	1	1	1	-1	1	-1	1	1	1	1	-1	-1	1	-1	-1	-1	1	-1	1

Supplementary Table 40: Weights of neuron 1 in 3-class 3-bit 28×28 image classification.

**Index**	**0**	**1**	**2**	**3**	**4**	**5**	**6**	**7**	**8**	**9**	**10**	**11**	**12**	**13**	**14**	**15**	**16**	**17**	**18**	**19**	**20**	**21**	**22**	**23**	**24**	**25**	**26**	**27**
**0**	-1	1	-1	1	-1	1	1	1	1	-1	1	-1	1	1	1	1	1	1	-1	1	-1	1	-1	1	-1	-1	1	1
**1**	-1	1	1	1	-1	1	1	-1	-1	-1	1	-1	-1	-1	1	-1	-1	-1	-1	1	-1	1	-1	1	1	1	-1	-1
**2**	1	1	-1	1	-1	-1	-1	-1	-1	-1	-1	-1	-1	-1	1	-1	-1	-1	-1	1	1	-1	1	-1	-1	-1	-1	-1
**3**	-1	1	1	1	1	-1	1	-1	1	1	-1	-1	-1	-1	1	-1	1	-1	1	-1	-1	-1	-1	-1	-1	-1	1	-1
**4**	-1	1	1	1	1	1	1	-1	1	1	-1	-1	-1	-1	1	1	-1	-1	-1	1	1	1	1	1	1	-1	1	1
**5**	-1	1	1	1	1	1	1	-1	-1	-1	-1	1	-1	1	-1	-1	-1	1	1	-1	1	1	1	1	1	-1	1	-1
**6**	1	1	-1	1	1	-1	-1	-1	-1	1	1	-1	1	1	-1	-1	1	1	-1	1	1	1	1	1	1	-1	-1	-1
**7**	1	1	-1	-1	1	-1	-1	1	-1	1	-1	-1	1	1	-1	1	-1	1	1	1	1	1	-1	-1	-1	-1	1	-1
**8**	-1	-1	-1	1	1	-1	-1	-1	-1	-1	-1	1	1	-1	-1	1	1	1	1	1	1	-1	-1	-1	-1	-1	-1	1
**9**	1	1	-1	-1	1	-1	-1	1	1	1	-1	1	-1	1	1	1	-1	1	1	-1	1	1	-1	-1	-1	-1	-1	1
**10**	-1	1	-1	-1	-1	-1	-1	1	-1	-1	-1	-1	1	1	1	1	1	-1	1	1	-1	-1	-1	-1	1	-1	-1	1
**11**	-1	-1	1	-1	-1	-1	-1	-1	1	-1	1	1	1	1	1	1	1	-1	-1	1	-1	1	1	1	-1	-1	-1	1
**12**	-1	-1	1	1	-1	1	-1	1	-1	-1	-1	-1	1	1	1	1	-1	-1	-1	1	1	-1	1	1	-1	-1	-1	-1
**13**	-1	1	-1	-1	-1	1	1	1	1	1	-1	-1	1	1	1	1	1	-1	-1	-1	1	1	1	-1	-1	-1	-1	-1
**14**	-1	1	1	-1	-1	1	-1	1	1	-1	-1	-1	1	1	1	1	1	-1	-1	-1	1	-1	1	1	-1	-1	-1	-1
**15**	-1	1	-1	-1	-1	-1	1	-1	-1	-1	-1	-1	1	1	1	1	-1	-1	-1	-1	1	-1	-1	1	-1	-1	-1	-1
**16**	1	1	1	-1	-1	-1	-1	1	-1	-1	-1	-1	1	1	1	1	-1	-1	-1	-1	-1	1	-1	1	-1	-1	-1	-1
**17**	-1	-1	-1	-1	-1	-1	1	-1	1	-1	-1	-1	-1	1	1	1	-1	-1	-1	-1	-1	-1	-1	-1	-1	-1	-1	-1
**18**	1	1	-1	-1	-1	-1	-1	-1	-1	-1	-1	-1	-1	1	1	1	-1	-1	-1	-1	1	1	-1	-1	-1	-1	-1	-1
**19**	1	-1	-1	1	-1	-1	-1	-1	1	1	-1	-1	-1	1	-1	-1	1	1	-1	1	1	-1	-1	1	-1	-1	-1	1
**20**	-1	-1	-1	-1	-1	-1	1	-1	-1	-1	1	1	1	-1	-1	-1	-1	-1	1	-1	-1	1	-1	-1	-1	-1	1	1
**21**	-1	1	1	-1	-1	-1	1	-1	1	-1	1	-1	-1	1	-1	1	-1	-1	-1	-1	-1	-1	1	-1	-1	1	-1	1
**22**	-1	1	-1	-1	-1	1	-1	-1	-1	1	-1	-1	-1	-1	-1	-1	-1	-1	-1	-1	-1	1	-1	-1	1	1	1	1
**23**	1	1	-1	-1	-1	1	-1	-1	1	-1	1	-1	1	-1	1	1	1	1	-1	-1	-1	-1	-1	1	-1	-1	1	-1
**24**	-1	1	-1	1	-1	1	1	-1	1	-1	-1	1	-1	-1	-1	-1	-1	-1	-1	-1	-1	-1	-1	-1	-1	-1	1	-1
**25**	1	-1	1	-1	-1	1	1	-1	1	1	-1	-1	1	-1	-1	-1	-1	-1	-1	-1	-1	-1	-1	1	-1	-1	-1	-1
**26**	1	-1	-1	1	-1	1	1	1	1	1	1	1	-1	1	1	1	1	-1	-1	1	1	1	-1	-1	-1	-1	-1	1
**27**	1	1	1	-1	-1	1	1	1	-1	1	-1	-1	1	1	1	-1	-1	1	1	-1	1	-1	1	-1	1	1	1	-1

Supplementary Table 41: Weights of neuron 2 in 3-class 3-bit 28×28 image classification.

**Index**	**0**	**1**	**2**	**3**	**4**	**5**	**6**	**7**	**8**	**9**	**10**	**11**	**12**	**13**	**14**	**15**	**16**	**17**	**18**	**19**	**20**	**21**	**22**	**23**	**24**	**25**	**26**	**27**
**0**	1	-1	-1	-1	-1	-1	1	-1	-1	1	1	-1	1	-1	-1	-1	-1	1	-1	-1	-1	-1	-1	-1	-1	1	1	-1
**1**	1	1	1	1	1	-1	-1	1	1	1	-1	-1	-1	-1	1	-1	1	1	1	-1	1	-1	-1	-1	-1	-1	1	1
**2**	-1	1	-1	-1	-1	-1	-1	1	1	1	1	1	1	1	1	1	1	1	1	-1	-1	1	-1	1	1	-1	1	1
**3**	1	1	-1	-1	-1	1	1	-1	-1	1	1	1	1	1	1	1	1	1	1	1	-1	1	1	1	1	1	1	-1
**4**	-1	-1	-1	-1	-1	1	-1	1	1	-1	1	1	-1	-1	1	-1	-1	-1	-1	-1	-1	-1	-1	-1	-1	1	1	1
**5**	-1	-1	-1	-1	-1	1	-1	-1	1	1	1	1	1	1	-1	1	1	1	1	1	-1	-1	-1	-1	-1	1	-1	1
**6**	1	-1	1	1	-1	-1	1	1	1	1	-1	1	1	1	1	1	-1	1	-1	-1	-1	-1	-1	-1	-1	1	1	-1
**7**	1	1	1	1	1	1	-1	-1	1	-1	1	-1	-1	-1	1	-1	-1	-1	-1	-1	-1	1	1	-1	-1	1	-1	1
**8**	-1	1	1	1	1	1	1	1	1	1	1	1	1	-1	-1	1	-1	-1	-1	1	-1	-1	1	1	-1	1	1	1
**9**	1	-1	1	1	1	1	1	-1	1	1	1	1	-1	1	-1	-1	-1	1	-1	-1	1	1	1	1	1	-1	1	1
**10**	1	-1	1	1	1	1	1	-1	-1	1	-1	1	-1	-1	-1	-1	1	1	-1	-1	-1	1	-1	-1	-1	1	1	-1
**11**	-1	-1	-1	1	1	1	-1	-1	1	-1	-1	-1	-1	-1	-1	-1	-1	-1	-1	1	-1	-1	-1	-1	-1	1	1	-1
**12**	1	-1	-1	1	-1	-1	-1	-1	-1	-1	-1	-1	-1	-1	-1	-1	1	1	-1	-1	-1	-1	-1	-1	-1	1	1	1
**13**	-1	-1	-1	1	-1	-1	-1	-1	-1	-1	-1	1	-1	-1	-1	-1	1	1	-1	1	-1	-1	-1	-1	1	1	1	1
**14**	1	-1	-1	1	-1	-1	-1	-1	-1	-1	1	1	1	-1	1	1	1	1	1	1	-1	-1	-1	-1	1	1	1	1
**15**	1	1	-1	1	-1	-1	-1	-1	-1	-1	1	1	1	1	1	1	1	1	1	1	1	1	-1	-1	1	1	1	1
**16**	1	-1	1	1	1	1	1	1	-1	-1	1	1	1	1	-1	1	1	1	1	1	-1	1	-1	1	1	1	1	1
**17**	-1	1	1	1	1	1	1	1	1	1	1	1	1	1	1	-1	1	1	1	-1	1	-1	-1	1	1	1	1	1
**18**	-1	-1	1	1	1	1	1	1	-1	1	1	1	1	1	1	1	1	1	1	-1	1	1	1	1	1	1	1	1
**19**	-1	-1	1	1	1	1	1	1	1	1	1	1	1	1	-1	1	1	1	1	1	1	1	-1	1	1	1	1	1
**20**	-1	-1	1	-1	1	1	1	-1	-1	-1	-1	-1	-1	-1	-1	1	1	1	1	1	1	1	-1	1	1	1	1	-1
**21**	1	-1	1	1	1	1	1	1	1	-1	1	-1	1	1	1	1	1	1	1	1	1	1	1	1	1	-1	1	-1
**22**	1	1	1	1	1	-1	1	1	1	1	-1	1	1	-1	-1	-1	1	1	1	1	1	1	1	1	-1	1	-1	-1
**23**	1	-1	1	1	1	1	1	-1	1	-1	1	-1	-1	-1	-1	-1	-1	1	1	1	1	1	1	1	1	1	-1	1
**24**	1	-1	-1	-1	1	-1	-1	-1	-1	1	-1	1	-1	1	-1	1	1	1	1	1	1	1	1	1	1	1	1	-1
**25**	1	1	-1	1	1	-1	1	1	-1	-1	1	1	1	1	1	1	1	1	1	1	1	1	1	-1	1	1	1	1
**26**	-1	1	1	-1	-1	-1	-1	-1	-1	1	-1	-1	1	-1	-1	-1	-1	1	-1	-1	1	1	-1	-1	1	1	1	-1
**27**	1	-1	1	1	-1	-1	-1	-1	1	-1	-1	1	1	1	-1	1	-1	-1	1	-1	-1	-1	-1	1	-1	1	-1	1

Supplementary References

[1] M. Alistar and U. Gaudenz, "Opendrop: An integrated do-it-yourself platform for personal use of biochips," *Bioengineering*, vol. 4, no. 2, p. 45, 2017.
