# Supplementary material for: Digital circuits and neural networks based on acid-base chemistry implemented by robotic fluid handling
Source: Nat Commun. 2023 Jan 30;14:496. doi: 10.1038/s41467-023-36206-8 (PMC9887006; doi:10.1038/s41467-023-36206-8)
Supplement: Supplementary file 3 — Description of Additional Supplementary Files [file 41467_2023_36206_MOESM3_ESM.pdf]

Description of Additional Supplementary Files

Supplementary Movie 1 – Classification of 8x8 Image.mp4: Walkthrough of running the 8x8 image classification using the acid-base model.

Supplementary Movie 2 – AND Gate on microfluidic board.mp4: Demonstrating AND-gate implementation on a the microfluidic board OpenDrop v4.
